# Placental Pathology, Perinatal Death, Neonatal Outcome, and Neurological Development: A Systematic Review

**DOI:** 10.1371/journal.pone.0089419

**Published:** 2014-02-25

**Authors:** Annemiek M. Roescher, Albert Timmer, Jan Jaap H. M. Erwich, Arend F. Bos

**Affiliations:** 1 Division of Neonatology, Beatrix Children's Hospital, University of Groningen, University Medical Center, Groningen, the Netherlands; 2 Department of Pathology and Medical Biology, University of Groningen, University Medical Center, Groningen, the Netherlands; 3 Department of Obstetrics and Gynecology, University of Groningen, University Medical Center, Groningen, the Netherlands; Xavier Bichat Medical School, INSERM-CNRS - Université Paris Diderot, France

## Abstract

**Background:**

The placenta plays a crucial role during pregnancy for growth and development of the fetus. Less than optimal placental performance may result in morbidity or even mortality of both mother and fetus. Awareness among pediatricians, however, of the benefit of placental findings for neonatal care, is limited.

**Objectives:**

To provide a systematic overview of the relation between placental lesions and neonatal outcome.

**Data sources:**

Pubmed database, reference lists of selected publications and important research groups in the field.

**Study appraisal and synthesis methods:**

We systematically searched the Pubmed database for literature on the relation between placental lesions and fetal and neonatal mortality, neonatal morbidity and neurological outcome. We conducted three separate searches starting with a search for placental pathology and fetal and neonatal mortality, followed by placental pathology and neonatal morbidity, and finally placental pathology and neurological development. We limited our search to full-text articles published in English from January 1995 to October 2013. We refined our search results by selecting the appropriate articles from the ones found during the initial searches. The first selection was based on the title, the second on the abstract, and the third on the full article. The quality of the selected articles was determined by using the Newcastle-Ottawa Quality Assessment Scale.

**Results:**

Placental lesions are one of the main causes of fetal death, where placental lesions consistent with maternal vascular underperfusion are most important. Several neonatal problems are also associated with placental lesions, whereby ascending intrauterine infection (with a fetal component) and fetal thrombotic vasculopathy constitute the greatest problem.

**Conclusions:**

The placenta plays a key role in fetal and neonatal mortality, morbidity, and outcome. Pediatricians should make an effort to obtain the results of placental examinations.

## Introduction

The placenta is the organ that links mother and fetus during pregnancy. It plays a crucial role in fetal growth and development by enabling the exchange of nutrients and oxygen from the mother to the fetus and removing fetal waste products.[1] The placenta is an endocrine organ, a site of synthesis and selective transport of hormones and neurotransmitters. In addition, the placenta forms a barrier to toxins and infective organisms.[2], [3] In recent years, findings based on placental lesions have contributed to a better understanding of how the placenta functions. Less than optimal placental performance may result in morbidity or even mortality of both mother and fetus. Indeed, there are indications that placental lesions are the main cause of fetal death.[4] It is also becoming increasingly clear that impaired placental functioning can have major implications for the live-born infant. Awareness among pediatricians, however, of the benefit of placental findings for neonatal care, is limited. Usually, the results of placental examinations are only reported back to the obstetrician instead of also passing it on to the pediatrician. In our opinion, this is a missed opportunity. Information on placental lesions can often be helpful towards explaining an abnormal neonatal outcome and might have consequences for treatment.

This article provides a systematic review of the relation between placental lesions and neonatal mortality, morbidity, and neurological development. We summarized the literature published on this topic during the past 18 years. Our hypothesis is that placental examination provides useful information about the pathophysiological mechanisms that lead to neonatal mortality and morbidity. Should this prove to be the case, this information is important for the pediatrician who should, therefore, be aware of and take into consideration the placental findings of their patients.

## Methods

### Literature search

This systematic review was conducted following the PRISMA guidelines for systematic reviews. A registered systematic review protocol is not available. Two independent researchers (AMR and AFB) searched the PubMed database for literature on the relation between placental lesions and perinatal mortality, neonatal morbidity, and neurological development. We limited our search to full-text articles published in English from January 1^st^ 1995 to October 31^st^ 2013. We conducted three separate searches starting with a search for placental lesions and fetal and neonatal mortality, followed by placental lesions and neonatal morbidity, and finally placental lesions and neurological development.

For the search on placental lesions and fetal and neonatal mortality, we used the terms (“placental pathology” AND “fetal death”) *OR* (“placental pathology” AND “stillbirth”) *OR* (“placental” AND “causes” AND “stillbirth”) *OR* (“placental pathology” AND “mortality”).

For the search on placental lesions and neonatal morbidity, we used the terms (“placental pathology” AND “morbidity”) *OR* (“placental pathology” AND “neonatal outcome”) *OR* (“placental lesions” AND “morbidity”) *OR* (“placental lesions” AND “neonatal outcome”) *OR* (“placenta” AND “neonatal implications”) *OR* (“placental” AND “lesions” AND “risk factor”).

For the search on placental lesions and neurological development, we used the terms (“placental pathology” AND “neurological”) *OR* (“placental pathology” AND “neurologic”) *OR* (“placental pathology” AND “cerebral palsy”) *OR* (“placental” AND “neurodevelopmental outcome”) *OR* (“placental pathology” AND “follow up”).

Subsequently, we refined our search results by selecting the appropriate articles from the ones found during the initial searches in three stages. The first selection was based on the title, the second on the abstract, and the third on the full-text article. Review articles on the subject of placental lesions and outcome were indicated as background articles. We did not use these articles in the tables, but we did use them in the text of our article. We were mainly interested in single births, therefore articles focusing primary on multiple births were excluded. In addition to the database search, we screened the reference lists of the selected articles, and the publications of important research groups in the field.

### Quality assessment

We assessed the quality of all the selected studies by means of the Newcastle-Ottawa Quality Assessment Scale for cohort and case-control studies. This assessment scale consists of three parts. For cohort studies these parts include selection, comparability, and outcome, for case-control studies selection, comparability, and exposure. The selection part consists of 4 items, with a maximum of 1 point per item. The comparability part has 1 item, with a maximum of 2 points for this item. Both the outcome and exposure parts consist of 3 items, with a maximum of 1 point per item. This provides a score, ranging from 0–9 points, with 9 points for the highest quality.

## Results

**Figure 1:**
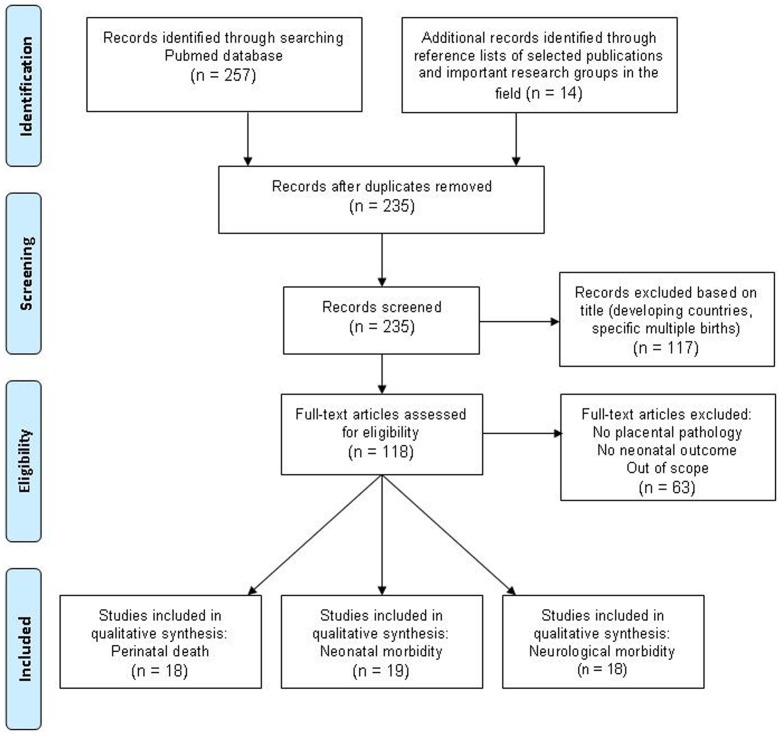


Our first search for placental lesions and perinatal mortality resulted in 135 articles. The second search for placental lesions and neonatal morbidity resulted in 55 articles. Our third search for placental lesions and neurological outcome produced 67 articles. After removing duplicates, we had a total of 221 articles. We excluded 117 articles based on their titles. Reasons for exclusion were studies with patient populations from developing countries or studies focusing on multiple births. Abstracts or full-text articles were assessed of the remaining 104 articles. Sixty-three articles were additionally excluded for the following reasons: no placental pathology performed, no neonatal outcome, and the studies being out of scope. By analyzing the reference lists of the remaining 41 articles, and screening publications from important research groups in the field, we additionally included 14 articles. Finally, 55 studies were included in our systematic review (Figure 1), i.e. 18 studies on perinatal death [4]–[21], 19 on neonatal morbidity [22]–[40], and 18 on neurological outcome.[41]–[58] Characteristics and the quality assessment scores of these 55 articles are presented in Tables 1–3.

**Table 1:** Description of selected studies perinatal mortality.

*Reference*	*Country*	*Study design*	*Study population*	*Study period*	*Sample size*	*Blinding placental examiner*	*Definition placental lesions*	*Corrected for confounders*	*Quality assessment Selection 4pt*	*Quality assessment Comparability 2pt*	*Quality assessment Outcome/exposure^a^ 3pt*	*Quality assessment Total 9pt*
Incerpi et al. (1998) [5]	USA	Cohort Retrospective Single-center	Stillbirths >20 wk GA, >500 g BW	1990–1994	745	NS	N	N	2	0	3	5
Ogunyemi et al. (1998) [6]	USA	Case-control Retrospective Single-center	Stillbirths ≥25 wk GA. Case: stillbirth	1985–1995	115 cases, 193 controls	N	Y	Y	3	2	2	7
Galan-Roosen et al. (2002) [7]	The Netherlands	Descriptive Prospective Multi-center	Stillbirths + neonatal death, >500 g BW	1983–1992	151 stillbirths, 88 neonatal death	N	N	N	4	0	2	6
Horn et al. (2004) [8]	Germany	Cohort Retrospective Single-center	Stillbirth ≥22 wk-<43 wk GA, ≥500 g BW	NS	310	N	N	N	3	0	3	6
Locatelli et al. (2005) [9]	Italy	Cohort Retrospective Single-center	Live born / neonatal death <750 g BW	1998–2002	59	Y	N	Y	3	2	2	7
Burke et al. (2007) [10]	Australia	ObservationalRetrospectiveMulti-center	Intrapartum death, all GA	NS	20	N	N	N	4	0	3	7
Zanconato et al. (2007) [11]	Italy	Cohort Retrospective Single-center	Stillbirth ≥22 wk GA, ≥500 g BW	2000–2006	59	N	N	N	4	0	2	6
Vergani et al. (2008) [12]	Italy	Cohort Retrospective Single-center	Stillbirth ≥22 wk GA, ≥500 g BW	1995–2007	154	N	N	N	4	0	3	7
Heazell et al. (2009) [13]	UK	Cohort Retrospective Single-center	Stillbirths	2006–2007	71	N	N	N	3	0	3	6
Kidron et al. (2009) [14]	Israel	Cohort Retrospective Single-center	Stillbirth 23–40 wk GA. Singletons	1994–2005	120	N	Y	N	4	0	3	7
Korteweg et al. (2009) [4]	The Netherlands	Cohort Prospective Multi-center	Antepartum death >20 wk GA	2002–2006	750	N	Y	N	4	0	3	7
Bonetti et al. (2011) [15]	Italy	Cohort Retrospective Single-center	Stillbirth ≥22 wk GA, ≥500 g BW	2000–2004	132	N	N	N	3	0	3	6
Tellefsen et al. (2011) [16]	Norway	Cohort RetroscpectiveSingle-center	Perinatal death ≥22 wk GA –7d post partum	2004–2008	104	N	N	N	4	0	3	7
VanderWielen et al. (2011) [17]	USA	Cohort Prospective Multi-center	Perinatal death + terminated pregnancies	NS	≥20 wk 330, ≤20 wk 73, 24 h pp 13	N	N	N	4	0	3	7
The stillbirth collaborative research writing group (2011) [18]	USA	Cohort Prospective Multi-center	Stillbirth ≥20 wk GA +18–19 wk GA if GA was uncertain	2006–2008	512	N	N	Y	4	1	3	8
Korteweg et al. (2012) [19]	The Netherlands	Cohort Prospective Multi-center	Stillbirth ≥20 wk GA	2002–2008	1025	N	N	N	3	0	3	6
Helgadottir et al (2013) [20]	Norway	Case-control Retrospective Multi-center	Stillbirth ≥22 wk GA, ≥500 g BW	1990–2003	377 cases, 1215 controls	Y	N	Y	2	2	1	5
Bring et al (2013) [21]	Sweden	Cohort study Prospective Multi-center	Stillbirth ≥22 wk GA	1998–2009	1089	NS	N	N	3	0	3	6

a: ‘outcome’ for cohort studies, ‘exposure’ for case-control studies.

Abbreviations: wk - weeks; GA - gestational age; BW - birth weight; NS - not stated; pp - post partum

**Table 2:** Description of selected studies neonatal morbidity.

*Reference*	*Country*	*Study design*	*Study population*	*Study period*	*Sample size*	*Blinding placental examiner*	*Definitions placental lesions*	*Corrected for GA*	*Quality assessment Selection 4pt*	*Quality assessment Comparability 2pt*	*Quality assessment Outcome/exposure^a^ 3pt*	*Quality assessment Total 9pt*
Beebe et al. (1996) [22]	USA	Cohort Retrospective Single-center	High risk population, all GA	1989–1992	1252	Y	Y	Y	3	2	2	7
Watterberg et al. (1996) [23]	USA	Case-control Prospective Single-center	Intubated infants <2000 gram. Case: RDS	1987–1989	38 cases, 15 controls	Y	Y	N	3	0	3	6
Baergen et al (2001) [24]	USA	Case-control Retrospective Single-center	All GA. Case: ELUC	1977–1995	926 cases, 200 controls	Y	Y	N	3	2	3	8
Redline et al. (2002) [25]	USA	Cohort Retrospective Single-center	VLBW infants <32 wk GA	1995–1997	371	Y	reference previous article	Y	3	2	3	8
Ogunyemi et al. (2003) [26]	USA	Cohort Retrospective Single-center	Preterm infants 24-32 wk GA	1992–2000	774	NS	Y	Y	4	2	3	9
Ariel et al. (2004) [27]	Israel	Cohort Prospective Single-center	Infants from pregnancies with preeclampsia, placental abruption or IUGR	NS	64	Y	Y	N	3	0	3	6
Holcroft et al (2004) [28]	USA	Cohort Retrospective Single-center	Preterm infants admitted NICU <34 wk GA	1999–2002	259	NS	Y	Y	4	2	3	9
Richardson et al. (2006) [29]	Canada	Cohort Retrospective Single-center	Preterm infants 25-34 wk GA	1995–2003	660	NS	Y	Y	3	2	3	8
Mehta et al. (2006) [30]	USA	Cohort Retrospective Single-center	Preterm infants admitted NICU ≤34 wk GA	1999–2001	165	Y	N	Y	3	1	2	6
de Laat et al. (2006) [31]	The Netherlands	Case-control Prospective Single-center	All GA. Cases: overcoiling / undercoiling UC	2002–2003	885	Y	Y	Y	3	1	3	7
Beaudet et al. (2007) [32]	Canada	Cohort Retrospective Single-center	NICU population placental pathology report available	1996–1997	1296	Y	NS	Y	3	2	3	8
Dix et al. (2010) [33]	Switzerland	Case-control Retrospective Single-center	Infants with NEC^b^, all GA. Case: NEC	1994–2005	77 cases. 769 controls	NS	Y	Subanalyses GA	2	0	3	5
Saleemuddin et al. (2010) [34]	USA	Case-control Retrospective Single-center	Infants with FTV, all GA. Case: FTV	1990–2007	113 cases. 216 controls	Y	Y	Y	3	2	3	8
Wintermark et al. (2010) [35]	Canada	Cohort Prospective Single-center	Infants with HIE undergoing induced hypothermia ≥36 wk GA	NS	23	Y	Y	N	4	0	3	7
Moscuzza et al. (2011) [36]	Italy	Cohort study Retrospective Single-center	NICU population placental pathology report available	2007	122	NS	N	N	2	0	3	5
Sato et al. (2011) [37]	Japan	Cohort Retrospective Single-center	NICU population <30 wk GA	2000–2008	302	NS	Y	Y	3	1	3	7
Roescher et al. (2011) [38]	The Netherlands	Cohort Retrospective Single-center	NICU population <32 wk GA	2006	40	Y	N	N	4	0	3	7
Chen et al. (2011) [39]	USA	Cohort Prospective Multi-center	ELGAN 23-27 wk GA	2002–2004	1064	Y	Y	Y	4	2	3	9
Perrone et al. (2012) [40]	Italy	Cohort Prospective Single-center	Preterm infants <32 wk GA	2008–2001	105	NS	Y	N	4	0	2	6

a: ‘outcome’ for cohort studies, ‘exposure’ for case-control studies.

b: Bell stage II and more.

Abbreviations: GA - gestational age; RDS - respiratory distress syndrome; ELUC - excessively long umbilical cord; VLBW - very low birth weight; NS - not stated; IUGR - intrauterine growth restriction; NICU - Neonatal Intensive Care Unit; UC - umbilical cord; NEC - necrotizing enterocolitis; FTV - fetal thrombotic vasculopathy; HIE - hypoxic ischemic encephalopathy; ELGAN - extremely low gestational age newborns.

**Table 3:** Description of selected studies neurological outcome.

*Reference*	*Country*	*Study design*	*Study population*	*Study period*	*Sample size*	*Blinding placental examiner*	*Definitions placental lesions*	*Corrected for GA*	*Quality assessment Selection 4pt*	*Quality assessment Comparability 2pt*	*Quality assessment Outcome/exposure^a^ 3pt*	*Quality assessment Total 9pt*
Redline et al. (1998) [41]	USA	Case-control Retrospective Single-center	NICU population <1500 g BW. Cases: NI at 20 m	1983–1991	60 cases, 59 controls	Y	Y	N	2	0	3	5
Redline et al. (2000) [42]	USA	Case-control Retrospective Single-center	Term infants. Cases: NI. Controls: meconium	1990–1997	40 cases, 176 controls	N	Y	Y	1	2	3	6
Viscardi et al. (2001) [43]	USA	Case-control Retrospective Single-center	NICU population all GA. Cases IUGR	1991–1996	94 cases, 145 controls	Y	Y	Y	2	2	3	7
Adams-Chapman et al. (2002) [44]	USA	Case-control Retrospective Single-center	NICU population <37 wk GA. Cases: MFI	1990–1998	21 cases, 42 controls	N	N	Y	3	2	2	7
McDonald et al. (2004) [45]	Ireland	Case-control Retrospective Single-center	Term infants. Cases: NE	1987–1998	93 cases, 387 controls	NS	Y	Y	3	2	2	7
Redline (2005) [46]	USA	Case-control Retrospective Single-center	Term infants. Cases: NI	NS	125 cases, 250 controls	N^b^	N	N	2	0	3	5
Polam et al. (2005) [47]	USA	Case-control Retrospective Single-center	NICU population 22–29 wk GA. Cases: AIUI	1997–2000	102 cases, 75 controls	Y	Y	Y	3	2	2	7
Redline et al. (2007) [48]	USA	Cohort Retrospective Single-center	NICU population ELBW infants <1 kg BW	1992–1995	129	Y	Y	Y	3	2	3	8
Reiman et al. (2008) [49]	Finland	Cohort Retrospective Single-center	Preterm infants <32 wk GA or ≤1500 g BW	2002–2006	121	Y	Y	Y	3	1	3	7
Suppiej et al. (2008) [50]	Italy	Cohort Retrospective Single-center	NICU population <32 wk GA	1998–2001	104	NS	N	N	2	0	1	3
Chau et al. (2009) [51]	Canada	Cohort Prospective Single-center	Preterm infants 24–32 wk GA	2006–2008	92	NS	N	N	3	2	3	8
Leviton et al. (2010) [52]	USA	Cohort Prospective Multicenter	ELGAN <28 wk GA	2002–2004	1246	NS	N	Y	4	1	3	8
Elbers et al. (2011) [53]	Canada	Cohort Retrospective Multi-center	Term + late preterm ≥34 wk GA. All neonatal stroke	1992–2006	12	NS	Y	N	2	0	3	5
Rovira et al. (2011) [54]	Spain	Cohort Retrospective Single-center	Preterm infants <32 wk GA, <1500 g BW	2002–2004	177	NS	Y	Y	4	2	3	9
Chang et al. (2011) [55]	Canada	Cohort Retrospective Single-center	IUFD 27–41 wk GA	2001–2007	37	Y	Y	Y	3	1	3	7
Blair et al. (2011) [56]	Australia	Case-control Prospective Multi-center	Late preterm+ term ≥35 wk GA. Cases: CP	1980–1995	445 cases, 497 controls	N	Y	N	4	0	1	5
Van Vliet et al. (2012) [57]	The Netherlands	Cohort Retrospective Single-center	Preterm infants ≤32 wk GA. AIUI+MVU	NS	72	Y	Y	Y	4	2	2	8
Hayes et al. (2012) [58]	Ireland	Case-control Retrospective / prospective Single-center	Term infants ≥36 wk GA. Cases: NE	2001–2008	141 cases, 309 controls	Y	N	Y	2	2	3	7

a: ‘outcome’ for cohort studies, ‘exposure’ for case-control studies.

b: Subgroup of placentas of both cases and controls were blinded re-reviewed.

Abbreviations: NICU - Neonatal Intensive Care Unit; BW - birth weight; NI - neurologic impairment; GA - gestational age; IUGR - intrauterine growth restriction; MFI - maternal floor infarction; NE - neonatal encephalopathy; AIUI -ascending intrauterine infection; ELBW - extremely low birth weight; ELGAN - extremely low gestational age newborns; IUFD - intrauterine fetal death; CP - cerebral palsy; MVU - maternal vascular underperfusion.

### Placental pathology

Examination of the placenta can reveal a wide range of pathologies. For good reproducibility it is necessary that placental lesions are well defined. Committees of the perinatal section of the Society for Pediatric Pathology have proposed definitions for maternal vascular underperfusion, fetal vascular obstructive lesions (fetal thrombotic vasculopathy), and the amniotic infection syndrome.[59]–[61] Definitions and descriptions of additional pathologies can be found in various textbooks on the pathology of the placenta.[62]–[67]

Since we acknowledge the fact that most pediatricians are unfamiliar with placental lesions and because a wide variety of terminology is used in the literature, we classified placental lesions according to the underlying pathology as previously proposed together with their pathological descriptions in Table 4.[35], [42], [59]–[61], [68]–[71]

**Table 4:** Overview of placental pathology relevant for understanding perinatal morbidity and mortality.

Diagnosis	Pathology and explanation	Outcome
Maternal vascular underperfusion (MVU)	Inadequate spiral artery remodeling or spiral artery pathology (decidual vasculopathy). Commonly seen in pregnancies complicated with pre-eclampsia. Expressed by parenchymal pathology such as placental hypoplasia, increased syncytial knots, villous agglutination, increased perivillous fibrin, distal villous hypoplasia, abnormal villous maturity, infarction, retroplacental hematoma. [59]	Fetal death [4], [8], [14], CP [48], [56]
Umbilical cord complications	Obstruction or disruption of the umbilical cord blood flow (e.g. umbilical cord prolapse, entanglement, knots, disrupted velamentous vessels, hyper/hypo-coiling). Can lead to fetal placental vascular stasis resulting in FTV. [35]	Fetal death [21], [31], fetal anomalies [24], asphyxia [31], [35], low Apgar score at 1–5 minutes [24], [31], RDS [24]
Fetal thrombotic vasculopathy (FTV)	Thrombosis, recent or remote, in the umbilical cord, chorionic plate or stem villus vessels and / or secondary degenerative pathology in the fetal vasculature distal to by thrombosis obliterated vessels (e.g. avascular chorionic villi). Expressed by hemorrhagic endovasculopathy, intimal fibrin cushions, fibromuscular hypertrophy, villous stromal-vascular karyorrhexis. [60]	Stillbirth [34], asphyxia [35], ↑ illness severity first 24h [38], NEC [32], [33], fetal cardiac abnormalities [34], ventriculomegaly [52], PVL [43], NI [41], [42], CP [46]
Distal villous immaturity / villous maturation defect	Maturation defect of the third trimester placenta characterized by enlarged chorionic villi with increased numbers of capillaries, macrophages, and fluid and decreased formation of vasculosyncytial membranes. As a result the diffusion distance between intervillous space and fetal capillaries is increased. [68]	Fetal death [4], asphyxia in diabetic pregnancy [68]
Villitis of unknown etiology (VUE)	Chronic lymphohistiocytic inflammation of the stem- and chorionic villi, with or without obliterative vasculopathy of stem villus vessels. [69]	Neonatal infection [22], NI [42], [46], NE [45], [58]
Ascending intrauterine infection (AIUI)	Acute chorioamnionitis and chorionitis (maternal response). The degree of severity can be staged and graded. [61]	Intrapartum death [10], Low Apgar score at 1–5 minute [22], [26], [35], neonatal infection [22], [26], [30], [36], ↓RDS [23], [29], [37], BPD [23], [26], [37], [40], ↑NEC [32], ROP [26], [36], [37], [39], IVH [26], [32], [36], [37], [47], ventriculomegaly [52], CP [52], NE [45], [58]
	Acute umbilical and chorionic vasculitis (fetal response). The degree of severity can be staged and graded. [61]	Low Apgar score at 1–5 minute [22], [26], [35], neonatal infection [22], [26], [30], [36], ↓RDS [29], [37], BPD [23], [26], [40], NEC [32], ROP[26], [36], [39], IVH [26], [30], [32], [36], [47], brain lesions [49], NI [42], [46], [54], NE [45], [58], disability in development at 2y [54]
Chronic deciduitis	Chronic lymphohistiocytic inflammation of placental villi. [70]	
Fetal hypoxia	Elevated nucleated red blood cells (NRBCs). Only rare NRBCs are normal after the first trimester. [42]	↑ illness severity first 24 h [38], NI [42]
	Chorangiosis. Diffuse increase in the number of villous capillaries [71]	

Abbreviations: CP - cerebral palsy; RDS - respiratory distress syndrome; NEC - necrotizing enterocolitis; PVL - periventricular leukomalacia; NI - neurological impairment; NE - neonatal encephalopathy; BPD - bronchopulmonary dysplasia; ROP - retinopathy of prematurity; IVH - intraventricular hemorrhage.

### Placental lesions and perinatal mortality

Perinatal mortality is defined as death during the perinatal period. In the 10^th^ Edition of the World Health Organization's International Classification of Diseases, the perinatal period is defined as death from 22 completed weeks of gestation up to 7 days after birth.[72] Fetal deaths form the largest group of perinatal mortality. In high-income countries one in every 200 infants that reaches 22 weeks' gestation or more, is stillborn.[73] Recently, the important role of the placenta in fetal deaths has become increasingly clear and several studies suggested that placental pathology is one of the main causes of fetal death (Table 5). This underscores the importance of examining the placenta, a fact sorely underestimated by obstetricians and general pathologists.[16]

**Table 5:** Results of selected studies on perinatal death.

*Placenta*	*Placental lesion*	*Ref.*	*Outcome measure: Perinatal death*	*Association found proportion* * */OR (95% CI)*	*No association found/ non placental*	*Remarks*
Placenta	**Not specified**	[11]	Stillbirth	Proportion 0.42 (0.31–0.55)	53% placenta negative	Placenta new insight
Placenta		[14]	Stillbirth	Proportion 0.33 (0.25–0.41)		Direct cause death
				Proportion 0.47 (0.38–0.56)		Major contributor
Placenta		[13]	Unexplained stillbirth	OR 0.17 (0.04–0.70)		After placental assessment stillbirth less likely to be unexplained
Placenta		[16]	Explanation perinatal death	Proportion 0.73 (0.64–0.81)	12% placenta no connection	Could explain death
				Proportion 0.51(0.41–0.66)	death	Cause explained by placental examination alone
Placenta		[12]	Stillbirth	Proportion 0.12–0.40 (0.08–0.48)		Different classification systems
Placenta		[4]	Stillbirth	Proportion 0.65 (0.61–0.69)		Placental lesions main cause fetal death
Placenta		[15]	Stillbirth	Proportion 0.22 (0.15–0.30)	51% no placental cause	Secondary main condition
Placenta		[17]	Stillbirth	Proportion 0.42 (0.37–0.47)	19.9% fetal, 13% maternal, 31.9% no cause	Proportion placental/cord causes stillbirth
Placenta		[18]	Stillbirth	Proportion 0.24 (0.20–0.28)	29.3% obstetric condition, 13.7% fetal abnormalities, 12.9% infection, 10.4% umbilical cord abnormalities	Placental second common cause stillbirth. Placenta main cause (26.1%) in antepartum deaths.
Placenta		[19]	Test determine cause death	Proportion 0.96 (0.94–0.97)	72.6% autopsy, 29.0% genetic analysis	Placental examination most valuable test for determination of cause stillbirth
Placenta		[9]	placental pathology in survivors and neonates who died		No differences in placental pathology between survivors and neonates who died.	
Placenta		[6]	Stillbirths	OR: 2.43 (1.12–5.26)		Positive placental pathology in 66% of stillbirths versus 44% in controls.
Placenta		[8]	Stillbirth	Proportion 0.62 (0.56–0.67)		Leading cause intrauterine death
Placenta		[5]	Evaluation Stillbirth	Proportion 0.30 (0.26–0.34)		Most important aspects stillbirth evaluation: placenta and autopsy
Placenta		[20]	Stillbirth	Proportion 0.50 (0.45–0.55)	19.4% unknown	Main cause of death. Placenta 18% associated condition death
Placenta	**Acute/subacute pathology**	[7]	Stillbirth + neonatal death	Proportion 0.32 (27–0.38)	23% congenital malformation, 16% infection, 8% prematurity, 7% unclassifiable	Most probable cause stillbirth
Placenta	**Chronic/progressive pathology**	[7]	Stillbirth + neonatal death	Proportion 0.21 (0.16–0.27)		Third most probable cause stillbirth
AIUI	**Ascending intrauterine infection**	[10]	Intrapartum death	Proportion 0.35 (0.18–0.57)	50% other (UC entanglement)	Proportion AIUI in intrapartum death
AIUI		[15]	Stillbirth	Proportion 0.23 (0.16–0.31)		Major relevant condition associated with death. Chorioamnionits diagnosed by bacterial cultures
MVU	**Maternal vascular underperfusion**	[14]	Stillbirth	Proportion 0.35 (0.27–0.44)		Direct/major contributor fetal death
MVU		[4]	Stillbirth	Proportion 0.34 (0.30–0.38)		Most important placental lesions in fetal death
MVU		[8]	Stillbirth	Proportion 0.38 (0.31–0.45)		Main contributor placental lesions to death
UC	**Umbilical cord lesions**	[15]	Stillbirth	Proportion 0.05 (0.02–0.10)		Proportion UC pathology in stillbirth
UC	Umbilical cord complication	[21]	Stillbirth	Proportion 0.08 (0.06–0.10)		Significant more in term stillbirths (9.75) compared to preterm stillbirths (6.4%)
UC	Undercoiling umbilical cord	[31]	Fetal death	OR 3.35 (1.48–7.63)		
UC	Overcoiling umbilical cord	[31]	Fetal death		Not significant. OR 2.43 (0.68–8.66)	
UC	Excessive long UC	[24]	Fetal/neonatal death		Not significant. OR 2.75 (0.65–36.14)	

*proportion placental lesions in perinatal death.

Abbreviations: AIUI - ascending intrauterine infection; MVU - maternal vascular underperfusion; UC - umbilical cord.

In 30% of the cases the cause of stillbirth is unknown.[73] In the remainder, i.e. the proportion of cases with known cause, most stillbirths are caused by placental lesions (12–65%, Table 5), followed by infections and umbilical cord abnormalities. [73] For lower gestational ages (GAs) (20 to 24 weeks), an unknown cause of death is most prominent, followed by placental lesions. At higher GAs, the relative importance of unknown causes decreases and placental causes increase.[73]

Placental pathology consistent with maternal vascular underperfusion is the main contributor to fetal death, ranging from 34 to 38 percent.[4], [8], [14] This is most prominent during the preterm period, in pregnancies complicated by hypertensive disorders, with a strong decline thereafter. During the term period, fetal death is mainly caused by developmental pathology of placenta parenchyma.[4] We can conclude that a pathological examination of the placenta is essential for clarifying causes of stillbirths.[5], [13], [14], [19]

The older classification systems for perinatal mortality did not address placental pathology, or specific placental lesions, as a separate group. Only in the more recent classification systems is placental pathology included as a cause of fetal death. In all recent stillbirth studies placental pathology is designated as one of the main causes of fetal death.[74], [75] The introduction of classification systems with placental pathology included as a separate group might be one of the reasons why recent studies identify placental pathology as one of the main causes of fetal death. Most of the placental lesions found in stillbirths, however, are also seen regularly after live, preterm or term, births.[76] The question arises whether placental lesions are also related to neonatal and neurological morbidity.

To summarize, in recent years the role of the placenta in fetal deaths has become increasingly clear. Placental pathology is one of the main causes of fetal death, with placental pathology consistent with maternal vascular underperfusion as the main contributor.

### Placental lesions and neonatal morbidity

It has been suggested that placental lesions are also associated with neonatal morbidity, but the association is less clear than for fetal mortality. Placental lesions are suggested to be associated with illness severity shortly after birth, and with a wide range of neonatal problems (Table 6).

**Table 6:** Results of selected studies on neonatal morbidity.

*Placental lesion*	*Placental lesion specified*	*Ref.*	*Outcome measure*	*Associations found OR (95% CI)*	*Association found other*	*No association found*	*Remarks*
AIUI	**Maternal + fetal response**	[22], [26], [35]	Low Apgar score	OR 1.7–2.1 (1.2–3.0)	Proportion AIUI: 0.35 95%CI (0.19–0.55) [35]		Apgar 1+5 minutes, asphyxia.
AIUI	(not specified)	[38]	Illness severity first 24h			No relation	
AIUI		[22], [26], [30], [32], [36]	Neonatal infection	OR 1.7–1.9 (1.2–3.0)	Effect size *r* = 0.31 [36]	No relation [32]	EOS + LOS + nosocomial infection
AIUI		[23], [26], [32]	RDS	OR 0.11 (0.02–0.63)		No relation [26], [32]	
AIUI		[23], [26], [32], [40]	BPD	OR 2.0–7.4 (1.20–31.16)		No relation [32]	
AIUI		[25]	BPD	OR 0.7 (0.4–0.9)			Unadjusted GA ns
AIUI		[26], [30], [32], [36], [40]	PDA	OR 1.7–5.53 (1.1–19.27)	Effect size *r* = 0.25 [36]	No relation [32], [30]	
AIUI		[26], [36], [39], [40]	ROP	OR 1.8–3.1 (1.02–9.5)	Effect size *r* = 0.52[36]	No relation [40]	In combination with micro-organisms [39]
AIUI		[26], [32], [40]	NEC	OR 3.80 (1.67–8.67)		No relation [26], [40]	
AIUI		[28]	Fetal metabolic acidosis			No relation	
AIUI		[22]	Liver disorders			No relation	
AIUI		[22]	Anomalies*			No relation	
AIUI	**Maternal response**	[28]	Fetal metabolic acidosis			No relation	
AIUI		[35]	Asphyxia		Proportion: 0.22 95% CI (0.10–0.42)		Proportion AIUI
		[37]	Sepsis			No relation	Stage AIUI
AIUI		[29], [37]	RDS	OR 0.6 (0.5–0.8)	Proportion RDS: 0.44 95% CI (0.35–0.53) [29]		Significant less than control group
AIUI		[29], [37]	BPD		59% with BPD had AIUI [37] Proportion BPD 0.26 95% CI (0.19–0.35) [29]	Adjusted for GA not significant [29]	
AIUI		[29], [37]	IVH		65.9% with IVH had AIUI [37]	No relation [29]	Adjusted for GA no relation [37]
AIUI		[37]	PDA			No relation	Stage AIUI
		[37]	ROP		62.9% with ROP had AIUI		Adjusted for GA no relation
AIUI		[37]	NEC			No relation	Stage AIUI
AIUI	**Fetal response**	[29]	RDS		Proportion 0.47 95% CI (0.40–0.55)		Significant less than control group
AIUI		[29]	BPD			No relation	
AIUI		[29], [30]	IVH	OR 1.95 (1.01–4.21)		No relation [29]	
MVU	**Maternal vascular underperfusion**	[38]	Illness severity first 24h			No relation	
MVU		[26]	Low Apgar score 1 min	OR 1.4–1.7 (1.02–2.5)			Apgar <7 (1+5 min)
MVU		[26], [32]	Neonatal infection			No relation	
MVU		[26], [32], [40]	NEC	OR 4 (1.7–9.2)		No relation [32], [40]	
MVU		[25], [26], [32], [40]	BPD			No relation	
MVU		[26], [32]	RDS			No relation	
MVU		[26], [32], [40]	PDA			No relation	
MVU		[26], [40]	ROP			No relation	
MVU	Placental infarction /abruption	[22]	Liver disorders	OR 2.2 (1.2–4.2)			Only with abruption
MVU		[22]	Low Apgar score 1 min			No relation	
MVU		[22]	Neonatal infection			No relation	
MVU		[22]	Anomalies*			No relation	
FTV	**Fetal thrombotic vasculopathy**	[34]	NRFHT	OR 3.01 (1.54–5.78)			
FTV		[34]	Fetal cardiac abnormalities	OR 8.02 (3.02–21.26)			
FTV		[34]	CNS abnormalities			No relation	
FTV		[35]	Asphyxia		Proportion: 0.26 95% CI (0.13–0.46)		Proportion FTV
FTV		[38]	Illness severity first 24h		Median scores illness severity significantly ↑		Higher illness severity
FTV		[32], [33]	NEC	OR 4.34–9.10 (1.80–15.08)			
FTV		[27]	Fetal thrombophilia			No relation	
FTV		[32]	Nosocomial infection			No relation	
FTV		[32]	RDS			No relation	
FTV		[32]	BPD			No relation	
FTV		[32]	PDA			No relation	
FTV		[32]	IVH			No relation	
VUE	**Villitis of unknown etiology**	[22]	Low Apgar score 1 min			No relation	
VUE		[38]	Illness severity first 24h			No relation	
VUE		[22]	Neonatal infection	OR 2.3 (1.1–5.1)			
VUE		[22]	Liver disorders			No relation	
VUE		[22]	Anomalies*			No relation	
Deciduitis	**Chronic deciduitis**	[38]	Illness severity first 24h			No relation	
Deciduitis		[32]	Nosocomial infection			No relation	
Deciduitis		[32]	RDS			No relation	
Deciduitis		[25], [30], [32]	BPD			No relation	
Deciduitis		[32]	NEC			No relation	
Deciduitis		[30], [32]	PDA			No relation	
Deciduitis		[30], [32]	IVH			No relation	
Deciduitis		[30]	ROP			No relation	
UC	**Umbilical cord lesions**	[35]	Asphyxia		Proportion UC: 0.39 95% CI (0.22–0.59)		Less in control group
UC	Excessively long umbilical cord	[24]	Apgar 1 min		Effect size *r* = −0.09		Lower Apgar scores
UC		[24]	Apgar 5 min		Effect size *r* = −0.07		Lower Apgar scores
UC		[24]	NRFHS	OR 4.91 (1.71–15.91)			
UC		[24]	Fetal anomalies	OR 13.10 (1.95–256.26)			
UC		[24]	Respiratory distress	OR 2.86 (1.09–8.17)			
UC	Undercoiling umbilical cord	[31]	Low Apgar 5 min	OR 3.14 (1.47–6.70)			
UC	Overcoiling umbilical cord	[31]	Asphyxia	OR 4.16 (1.30–13.36)			
Marker	**Elevated NRBCs**	[38]	Illness severity		Median scores illness severity significantly ↑		Higher illness severity
Marker		[36]	LOS			No relation	
Marker		[36]	PDA			No relation	
Marker		[36]	ROP			No relation	
Marker		[36]	IVH			No relation	
Marker	**Chorangiosis**	[22]	Low Apgar score 1 min			No relation	
Marker		[38]	Illness severity first 24h			No relation	
Marker		[22]	Neonatal infection			No relation	
Marker		[22]	Liver disorders			No relation	
Marker		[22]	Anomalies*			No relation	
Other	**Villus edema**	[32]	BPD	OR 1.46 (1.04–2.05)			
Other		[32]	Nosocomial infection			No relation	
Other		[32]	RDS			No relation	
Other		[32]	NEC			No relation	
Other		[32]	PDA			No relation	
Other	**Meconium staining**	[22]	Low Apgar score 1 min			No relation	
Other		[22], [32]	Neonatal infection			No relation	
Other		[32]	RDS			No relation	
Other		[32]	BPD			No relation	
Other		[32]	NEC			No relation	
Other		[32]	PDA	OR 0.18 (0.05–0.68)			
Other		[32]	IVH			No relation	
Other		[22]	Liver disorders			No relation	
Other		[22]	Anomalies			No relation	
Other	**Chorionic plate meconium**	[35]	Asphyxia	Proportion	0.30 95% CI (0.16–0.51)		
Other	**Coagulation related lesions**	[26]	NEC	OR 2.6 (1.13–6.00)			
Other		[26]	Low Apgar score			No relation	Apgar 1–5 minutes
Other		[26]	RDS			No relation	
Other		[26]	BPD			No relation	
Other		[26]	IVH			No relation	
Other		[26]	ROP			No relation	
Other		[26]	EOS			No relation	
Other		[26]	PDA			No relation	
Other	**Placental ischemic changes**	[22]	Neonatal infection	OR 0.54 (0.35–0.84)			
Other		[22]	Low Apgar score 1 min			No relation	
Other		[22]	Liver disorders			No relation	
Other		[22]	Anomalies*			No relation	

*Anomalies: notations of dysmorphia, hydrocephalus, Down syndrome.

Abbreviations: EOS - early onset sepsis; LOS - late onset sepsis; RDS - respiratory distress syndrome; BPD - bronchopulmonary dysplasia; GA - gestational age; PDA - patent duct arteriosus; ROP - retinopathy of prematurity; NEC - necrotizing enterocolitis; NRFHT - non-reassuring fetal heart tracing; CNS - central nervous system; NRFHS - non-reassuring fetal heart status.

Abbreviations placental lesions: AIUI - ascending intrauterine infection; MVU - maternal vascular underperfusion; FTV - fetal thrombotic vasculopathy; VUE - villitis of unknown etiology; UC - umbilical cord; NRBCs - elevated nucleated red blood cells.

Illness severity shortly after birth can be determined by the presence of asphyxia, Apgar scores during the first minutes after birth, and by several clinical variables during the first 24 hours after birth. Perinatal asphyxia is described to be associated with placental lesions affecting fetal vascular supply. These lesions were umbilical cord complications (disrupted velamentous vessels, cord tear, hypercoiled cord, cord hematoma), chorioamnionitis with fetal vasculitis, and fetal thrombotic vasculopathy.[31], [35] Low Apgar scores at 1 and 5 minutes are associated with ascending intrauterine infection and maternal vascular underperfusion.[22], [26] Higher illness severity during the first 24 hours after birth, determined by the Score of Neonatal Acute Physiology Perinatal Extension (SNAPPE), is associated with placental pathological findings of fetal thrombotic vasculopathy and elevated nucleated red blood cells (a sign of hypoxia).[38]

Lung development and neonatal respiratory problems, such as neonatal respiratory distress syndrome (RDS) and bronchopulmonary dysplasia (BPD), are associated with placental inflammation. There are indications that the incidence of RDS is reduced in infants exposed to chorioamnionitis (ORs 0.1–0.6, 95% CI: 0.02–0.8).[23], [29], [37], [77] This beneficial effect may be explained in several ways. It can be explained by advanced lung maturation in terms of an early elevation of interleukin-1 beta (IL–1β) in lung lavage fluid in the presence of chorioamnionitis, which stimulates the release of corticotrophin-releasing factor and corticotrophin.[78], [79] These hormones enhance the production of cortisol which results in accelerated lung maturation and, therefore, a decrease in the incidence of RDS.[80] Lung maturation is also explained with animal models of fetal inflammation. Chorioamnionitis in the fetal lung induces elevated IL-1, which in turn increases the amounts of surfactant proteins in parallel with increases in surfactant lipids in bronchoalveolar lavages. The lung mesenchymal tissue decreases, which increases the epithelial surface area and airspace volume of the lung. This results in a more mature lung structure that contains more surfactant, has increased compliance, and supports better gas exchange.[77], [81], [82]

Besides potentially a beneficial effect on lung function immediately after birth, an ascending intrauterine infection can also have a detrimental effect on the preterm lung, particularly in the long-term.[77] Chorioamnionitis can promote BPD, with ORs ranging from 2.0–7.4 (95% CI: 1.2–31.2).[23], [26], [37], [40], [77], [83] BPD results from multiple antenatal and postnatal factors (hits) contributing to disease progression.[84] Despite a healthier initial condition (less RDS), the pulmonary status worsens during the postnatal period.[83] This is explained by an increased susceptibility of the lung to postnatal injurious events (second hits).[83]–[86] Even so, the relation between respiratory problems and chorioamnionitis is difficult to assess, since it is confounded by a variety of prenatal factors.[85]

Necrotizing enterocolitis (NEC) is a challenging problem in the neonatal care of, mainly, preterm infants. The etiology of NEC is still poorly understood, but it is believed to be multifactorial.[87] Several studies found an association between NEC and placental lesions, in particular fetal vascular obstructive lesions (fetal thrombotic vasculopathy, congested villi, coagulation-related lesions) with ORs ranging from 2.6 to 9.10 (95% CI: 1.13–15.08).[26], [32], [33] The presence of ischemia has been proposed as an explanation for the etiology of NEC. Placental vasculopathy, which causes uteroplacental insufficiency, may cause fetal circulatory adaptive changes to hypoxia, which may result in bowel ischemia predisposing to NEC.[26]

Retinopathy of prematurity (ROP) is also associated with placental lesions, in particular with inflammatory lesions with ORs ranging from 1.8 to 3.1 (95% CI: 1.02–9.5). [26], [36], [37], [39], [88] ROP affects preterm infants and is caused by disorganized growth of retinal blood vessels which may result in scarring and retinal detachment. The etiology of ROP is likely to be a multihit phenomenon. At least part of the multihit is an inflammation-related pathogenesis, which is thought to be mediated by cytokines and growth factors present in the newborn's systemic circulation.[39] The severity of ROP also correlates positively with ascending intrauterine infection.[88]

Fetal cardiac abnormalities are also thought to be associated with placental lesions. A six-fold increase in fetal cardiac abnormalities is reported in the presence of fetal thrombotic vasculopathy.[34] The most common cardiac abnormalities found in its presence are ventricular and atrial septal defects, cardiomegaly, and coarctation of the aorta. It is hypothesized that the relation may be explained by a causal link between the two lesions.[34] The presence of one lesion may lead to the establishment of the other, through abnormal blood flow which serves as the common denominator. Another theory is that a common genetic variation underlies both placental fetal thrombotic vasculopathy and abnormal development of the heart.[34] This theory is supported by studies in mice which have shown placental and cardiac functions to be intimately linked, both through secretion of placental factors which affect maternal and fetal circulation and through genes which contribute to the development of both organ systems.[89] In addition, ascending intrauterine infection with both maternal and fetal response is associated with an increased risk for patent ductus arteriosus with ORs ranging from 1.7 to 5.53 (95% CI: 1.1–19.27).[26], [36], [40]

To summarize, the most important placental lesions in neonatal morbidity seem to be ascending intrauterine infection and fetal thrombotic vasculopathy. Nevertheless, caution is required in order to interpret these findings properly. Many studies on neonatal outcome only focus on infectious placental lesions and fetal thrombotic vasculopathy to the exclusion of other placental lesions. Thus there may be a bias towards these two lesions, because chorioamnionitis is a placental lesion well-known to both gynecologists and pediatricians. Even so, four of the larger studies including a wide range of placental lesions identified ascending intrauterine infection and fetal thrombotic vasculopathy as the most important placental finding with respect to neonatal morbidity.[22], [26], [32], [40] This may pave the way for early interventions serving to prevent morbidity. Before such interventions can be defined, however, detailed knowledge of the pathophysiological mechanisms that lead to neonatal morbidity is required.

### Placental lesions and neurological morbidity

Many prospective and retrospective studies have been conducted on placental lesions and neurological morbidity (Table 7). Some of the studies focused on early brain development, while others focused on neurological and functional outcome as determined by follow-up testing. However, it is difficult to conduct correlative studies between placenta lesions and neurologic or psychiatric outcomes in the child.[90] Neurological outcomes are not evident immediately after birth, but only long after most placentas have been discarded. Placentas, especially those of term infants, are not routinely sent to the pathologist for examination.[55], [90] Unless studied prospectively, infants whose placentas are examined, form a biased group.[90]

**Table 7:** Results of selected studies on neurological outcome.

*Placental lesion*	*Placental lesion specified*	*Ref.*	*Outcome measure*	*Associations found OR (95% CI)*	*Association found other*	*No association found*	*Remarks*
AIUI	**Maternal + fetal response**	[26], [32], [36], [40], [47]	IVH	OR 1.7–3.5 (1.2–23)	*r* * = 0.71[36]	No relation [40]	
AIUI	Not specified	[51]	WMI			No relation	Stage/grade AIUI also not associated WMI
AIUI		[43]	Ultrasound abnormalities			No relation	IVH, PVL, infarction
AIUI		[55]	Neuronal karyorrhexis or white matter gliosis		No data (*p*<0.05)		Neuropathology in stillbirths
AIUI		[47]	Neurodevelopment			No relation	Age: 12–24m BSID-II
AIUI		[50]	Speech abnormalities	OR: 5.1 (1.35–19.4)			18months
AIUI		[50]	Hearing loss	OR 11.6 (1.3–105.9)			18months
AIUI		[50]	Motor development			No relation	18months
AIUI	**Maternal response**	[29], [54]	IVH	OR 2.4 (1.0–5.6)		No relation [29]	Adjusted for GA not significant [54]
AIUI		[52]	Ventriculomegaly	OR 1.4–1.5 (1.01–2.4)			
AIUI		[48], [52], [54]	CP	OR 2.3–3.4 (1.1–7.4)		No relation [48], [54]	
AIUI		[45], [58]	Neonatal encephalopathy	OR 2.02 (1.16–3.74)	RRR 3.3 (1.1–10.4) [58]		Adjusted for confounders not significant [45]
AIUI		[49]	Brain lesions			No relation	IVH, cPVL, ventriculomegaly
AIUI		[41]	Neurolic impairment			No relation	VLBWI
AIUI		[54]	Motor abnormalities	OR 3.68 (0.95–14.28)			24 m Bayley-II or Brunet-Lezine scale
AIUI		[54]	Any grade disability			No relation	24months
AIUI		[54]	Speech abnormalities			No relation	24months
AIUI		[54]	Hearing loss			No relation	24months
AIUI		[48]	Neurocognitive function			No relation	ELBWI follow-up 8y
AIUI	**Fetal response**	[29], [30], [54]	IVH	OR 2.0–2.3 (1.0–5.5)		No relation [29]	Adjusted for GA not significant [54]
AIUI		[51]	WMI			No relation	
AIUI		[52]	Ventriculomegaly			No relation	OR 1.4 (0.9–2.2) [52]
AIUI		[48], [52], [54]	CP	OR 4.32 (0.91–20.44)		No relation [48], [52]	OR 1.7 (0.8–3.7) [52]
AIUI		[41], [42], [46]	Neurologic impairment	OR 2.9–13.2 (1.2–144)		No relation [41]	
AIUI		[45], [58]	Neonatal encephalopathy	OR 22.54 (11.07–45.91)	RRR 20.7–34.6 (1.8–232.9) [58]		
AIUI		[49]	Brain lesions	OR 2.46 (1.13–5.41)		Adjusted for GA not significant	IVH, cPVL, ventriculomegaly
AIUI		[54]	Moderate to severe disability	OR 4.08 (1.16–14.44)			24months
AIUI		[54]	Speech abnormalities	OR 2.89 (1.19–7.04)			24months
AIUI		[54]	Hearing loss			No relation	24months
AIUI		[48]	Neurocognitive function			No relation	ELBWI follow-up 8y
MVU	**Maternal vascular underperfusion**	[26], [32], [40]	IVH			No relation	
MVU		[52]	Ventriculomegaly	OR 0.5 (0.3–0.96)			
MVU		[45]	Neonatal encephalopathy	OR 3.86 (1.36–10.92)			
MVU		[53]	Neonatal stroke		Proportion 0.25 (0.09–0.53)		3 placentas of 12 infants with neonatal stroke
MVU		[41]	Neurologic impairment			No relation	VLBWI
MVU		[48], [52]	CP	OR 7.4–10.1 (1.6–46.3)		No relation [52]	OR 1.5 (0.3–6.6) [52]
MVU		[57]	Neurodevelopment 2y Bayley-II MDI + PDI		Cohen's *d*: 1.12 MDI	PDI no relation	Lower MDI scores compared to AIUI
MVU		[48], [57]	Neurodevelopment 7/8y			No relation	WISC, MABC, CBCL compared to AIUI
MVU	**Macroscopic placental infarct**	[56]	CP	OR 2.6 (1.23–5.57)			
FTV	**FTV**	[32]	IVH			No relation	
FTV		[52]	Ventriculomegaly	OR 2.1 (1.2–3.9)			
FTV		[43]	Ultrasound abnormalities	OR 5.41 (1.42–20.54)			IVH, PVL, infarction
FTV		[48], [52]	CP			No relation	OR 1.9 (0.8–4.3) [52]
FTV		[41], [42], [46]	Neurologic impairment	OR 3.7–9.2 (1.0–51)			Only chorionic plate thrombi [41]
VUE	**VUE**	[42], [46]	Neurologic impairment	OR 4.1–7.4 (1.3–17.9)		Adjusted for GA not significant [42]	With oblirative fetal vasculopathy [46]
VUE		[45], [58]	Neonatal encephalopathy	OR 2.11 (1.16–3.83	RRR 17.7 (5.0–60.8) [58]		Adjusted for confounders not significant [45]
VUE		[43]	Ultrasound abnormalities			No relation	IVH, PVL, infarction
		[55]	Neuronal karyorrhexis or white matter gliosis			No relation	Neuropathology in stillbirths
Deciduitis	**Deciduitis**	[32]	IVH			No relation	
MFI	**Maternal floor infarction**	[43], [44]	Ultrasound abnormalities			No relation	IVH, PVL, infarction
MFI		[44]	WMI	OR 3.7 (1.1–12.7)			
MFI		[44]	Neurodevelopment	OR 14 (2–163)			Age:22–29months
Marker	**Elevated NRBCs**	[36]	IVH			No relation	
Marker		[55]	Neuronal karyorrhexis or white matter gliosis			No relation	Neuropathology in stillbirths
Marker		[42]	Neurologic impairment	OR 22.3 (11–46)		Adjusted for GA not significant	
Marker	**Stressful intrauterine environment**	[53]	Neonatal stroke		Proportion 0.17 (0.05–0.45)		1 case ↑NRBCs and 1 case chorangiosis
Other	**Villus edema**	[48]	Neurocognitive function	OR 4.7 (1.1–19.2)			ELBWI follow-up 8y
Other		[41]	Neurologic impairment	OR 5.7 (1.5–21.0)			
Other		[45]	Neonatal encephalopathy	OR 4.63 (2.01–10.68)			
Other		[55]	Neuronal karyorrhexis		No data (*p*<0.05)		Neuropathology in stillbirths
Other		[30], [32]	IVH	OR 2.57–2.19 (1.01–6.58)			
Other	**Coagulation related lesions**	[26]	IVH			No relation	
Other	**Meconium staining**	[32]	IVH			No relation	
Other		[55]	Gliosis		No data (*p*<.05).		Neuropathology in stillbirths
Other	**Meconium-associated vascular necrosis**	[42], [46]	Neurologic impairment	OR 4.8–8.2 (2.0–29.0)		Adjusted for GA not significant.[42]	
Other	**Meconium phagocytosis**	[58]	Neontal encephalopathy		RRR 7.2–9.8 (2.3–42.4)		
Other	**Chorioamnionic hemosiderosis**	[42]	Neurologic impairment	OR 74.8 (6.3–894)			
Other	**Sudden catastrophic event**	[53]	Neonatal stroke		Proportion 0.42 (0.19–0.68)		Retroplacental hematoma and umbilical cord occlusion
Other	**Thrombo-inflammatory process**	[53]	Neonatal stroke		Proportion 0.5 (0.25–0.75)		Acute chorioamnionitis, chronic villitis, chorionic vessel thrombi, avascular villi

*r =  effect size.

Abbreviations: IVH- intraventricular hemorrhage; WMI - white matter injury; PVL - periventricular leukomalacia; BSID - Bayley scales of infant development; GA - gestational age; CP - cerebral palsy; cPVL – cystic periventricular leukomalacia; ELBWI - extremely low birth weight infant; VLBWI - very low birth weight infant; MDI - mental development index; PDI - psychomotor development index; WISC - Wechsler Intelligence Scale for Children; MABC - movement assessment battery for children; CBCL - Children Behavior Checklist.

Abbreviations placental lesions: AIUI - ascending intrauterine infection; MVU - maternal vascular underperfusion; FTV - fetal thrombotic vasculopathy; VUE - villitis of unknown etiology; MFI - maternal floor infarction; NRBCs - nucleated red blood cells.

It is thought that the pathogenesis of neurological impairment has an antenatal as well as an intra-partum component. An event weeks before delivery can result in a non-optimal fetal environment. This might result in lowering the threshold required for more recent events to cause brain injury. Placental lesions can be such an antenatal event.[41], [42], [45]

Regarding short-term neurological outcome of preterm infants in particular, most studies focused on white matter diseases (periventricular leukomalacia, PVL) and intraventricular hemorrhages (IVH). The results are inconsistent as far as the relation between these short term neurological outcomes and placental lesions is concerned. Several studies did find a relation between IVH and histological ascending intrauterine infection (maternal and fetal response) with ORs ranging from 1.7 to 2.2 (95% CI 1.01–23).[26], [30], [32], [36], [47] In addition, the severity of ascending intrauterine infection is significantly higher among infants with IVH.[37] After adjusting for gestational age, however, the severity of ascending intrauterine infection did not seem to affect the occurrence of IVH. Others were not able to find a relation between IVH and AIUI.[29], [40] There are no indications that other placental lesions are associated with IVH.

Regarding white matter injury, several studies failed to find a relation with histological AIUI (maternal and fetal response).[43], [51] Nevertheless, in a meta-analysis, ascending intrauterine infection (clinical and histological) was indicated as a risk factor for white matter injury in preterm infants, with a relative risk of approximately 2.1.[91] The authors hypothesized that elevated cytokine levels play a role in the etiology of white matter brain lesions. The reason for the inconsistency of the results may be ascribed to differences in adjusting for potential confounders. Wu et al.[91] explained the effect of adjusting for gestational age. Although gestational age appears to be a possible confounder, it may also lie directly in the causal pathway between maternal infection and cerebral palsy (CP). Chorioamnionitis is associated with preterm delivery, and low gestational age is in turn associated with a host of intrinsic vulnerabilities within the brain that have been implicated in the pathogenesis of cystic PVL and CP. Therefore, if low gestational age resulting from maternal infection in itself plays a direct role in the pathogenesis of CP, then adjusting for its effect will falsely diminish the observed association between chorioamnionitis and CP.[91]

Neonatal encephalopathy has mainly an antepartum, rather than an intrapartum, etiology. An important antepartum factor is placental pathology.[45], [58] Placental lesions consistent with fetal thrombotic vasculopathy (OR 4.63, 95% CI: 2.01–10.68) and AIUI with a fetal response (funisitis) (OR 22.54 95% CI: 11.07–45.91) are both associated with neonatal encephalopathy.[45], [58] Another less strongly associated placental lesion is accelerated villous maturation (disturbed uteroplacental flow) with an OR of 3.86 (95% CI: 1.36–10.92).[45]

Elbers et el. studied placental pathology in relation to neonatal stroke.[53] They systematically described their findings in twelve cases of neonatal stroke, ten of which had placental lesions. They found the following types of lesions: thromboinflammatory process in six cases, sudden catastrophic event in five cases, decreased placental reserve in three cases, and stressful intrauterine environment in two cases. They suggested that multiple risk factors are involved in neonatal stroke, and that placental pathology may be a contributing factor.[53]

The Extremely Low Gestational Age Newborns (ELGAN) investigators studied the predictive value of placental pathology in regard to white matter damage and later CP. They found histologic inflammation to be predictive of ventriculomegaly and diplegic CP, with ORs ranging from 1.4 to 1.5 (95% CI: 1.0–2.4) and ORs 2.3–3.4 (95% CI: 1.1–7.4), respectively. Placental inflammation was not predictive for echolucent lesions.[52] Also fetal thrombotic vasculopathy is found to be associated with CP. In the presence of FTV and CP, obstructive umbilical cord abnormalities have been identified. These umbilical cord abnormalities can lead to fetal placental vascular stasis resulting in fetal thrombotic vasculopathy.[42], [46], [92] Macroscopic examination of the placenta can also identify an increased risk of CP. Placental infarction thus identified is associated with an increased risk of the spastic quadriplegic subtype of CP (OR 2.6, 95% CI: 1.2–5.6).[56] The pathophysiological mechanism of placental infarction leading to CP is not clear. It is stated that because of the many functions and substantial functional reserve of the placenta, it cannot be assumed that placental infarction acts mainly by interference with gas exchange. A hypothesis is that, whatever the underlying process that harmed the vasculature of the placenta causing infarction, the same process may also have directly harmed either the fetal cerebral vasculature or the brain.[56]

Results on the association between placental pathology and long-term neurological outcome, including developmental tests and functional outcome, are also inconsistent between studies. In preterm infants it is thought that neurological impairment is associated with recent non-occlusive thrombi of the chorionic plate vessels in combination with chorioamnionitis and severe villous edema. Chorioamnionitis alone is not associated with neurological impairment.[41] This was attributed to the strong and consistent relationship between neurologic impairment and chorionic plate thrombi that occur only in placentas with chorioamnionitis.[41] Placental pathology consistent with maternal vascular underperfusion was also found to be a risk factor for neurological impairment, with ORs ranging from 7.4 to 10.1 (95% CI: 1.6–46.3).[48]

For term infants, Redline et al. reported an association between neurological impairment and ascending intrauterine infection with a fetal response (OR 2.9–13.2, 95% CI: 1.2–144).[42], [46] In addition to AIUI with a fetal response, they found that the following lesions are present significantly more often in placentas of infants with neurological impairment: meconium associated vascular necrosis, chorionic vessel thrombi, increased nucleated red blood cells (sign of fetal hypoxia), findings consistent with abruption placenta, diffuse chronic villitis, extensive avascular villi, diffuse chorioamnionic hemosiderosis, and perivillous fibrin.[42]

Neurodevelopmental outcome of preterm-born children at toddler age is also associated with ascending intrauterine infection with a fetal response (funisitis). In the presence of funisitis, a higher incidence of moderate to severe disability is present with an OR of 4.08 (95% CI: 1.16–14.44).[54] In addition, speech abnormalities and hearing loss are associated with AIUI (ORs 2.9–5.1, 95% CI: 1.2–19.4 and OR 11.6, 95% CI: 1.3–105.9, respectively. A study comparing neurodevelopmental outcome at two years of age between very preterm infants with maternal vascular underperfusion and very preterm infants with histological chorioamnionitis found poorer mental development in infants with maternal vascular underperfusion compared to infants with chorioamnionitis.[57]

Neurocognitive outcome of preterm-born children at school age is associated with villous edema (OR 4.7, 95% CI: 1.1–19.2). Lower scores on mental processing and on neuropsychological assessment are found in its presence. In this study, ascending intrauterine infection is not predictive of impaired neurodevelopmental outcome in the population as a whole, but a severe funisitis is associated with lower scores on neurocognitive tests in the subpopulations with ascending intrauterine infection.[48]

In summary, despite the difficulties in studying the relation between placental lesions and neurological morbidity, and the inconsistent results, some conclusions can be drawn. For those studies finding a relation with poor neurological outcome, the placental lesion is ascending intrauterine infection with a fetal response. Furthermore, in term infants a larger variety of placental lesions seem to be associated with poor neurological outcome compared to preterm infants. Knowledge on the pathophysiological mechanisms leading to long-term neurological deficits may lead to possible interventions to improve outcome. The fact that the placenta is available for histological examination immediately after birth and that it may reveal valuable information for pediatricians, leads to an early opportunity to intervene to the benefit, hopefully, of ill neonates.

## Discussion/Conclusion

The placenta plays a key role in fetal and neonatal mortality, morbidity, and outcome. Placental lesions are one of the main contributors to fetal death. In these cases placental lesions consistent with maternal vascular underperfusion are most important. Although less clear-cut, several neonatal problems are also associated with placental lesions. Regarding neonatal morbidity and neurological outcome, placental lesions with ascending intrauterine infection (with a fetal component) and fetal thrombotic vasculopathy, constitute the greatest problem.

To our surprise we noticed a difference in the description of placental lesions between studies on perinatal death and studies on neonatal outcome. The majority of studies on placental pathology and stillbirth only focus on the presence or absence of placental lesions in general, but they do not examine the relation between specific placental lesions and stillbirth. Studies concerning placental lesions and neonatal or neurological outcome do specify the lesions, finding several relations between specific placental lesions and outcome. Characterizing placental lesions in more detail in stillbirth studies may provide additional information concerning the cause of death.

Most studies report on associations between placental lesions and outcomes but this does not necessarily reflect a causal relation. There is still need to clarify pathophysiological mechanisms. One of these proposed mechanisms include gene-environment interactions.[92] Placental lesions might already have their onset early in pregnancy, due to changes in placental genes, leading to epigenetic alterations. Causes for these placental epigenetic changes may include a non optimal intrauterine environment, due to a maternal disease or adverse insults to the intrauterine environment.[93] This may in turn cause placental dysfunction and hence adverse neonatal outcome. We thus have to take into account that multiple interactions from maternal, placental, and fetal health play a role in the etiology of perinatal death and neonatal morbidity. Future research must consider statistical tools to better address interactions among these multiple variables, such as a mixed-effect regression analyses for example.

There are several limitations to our systematic review. Firstly, there is a potential risk of publication bias. Studies finding negative results regarding placental lesions and outcome might not be published. This may lead to an overestimation of associations between placental lesions and outcomes. Secondly, we included studies from the past 18 years. Earlier studies might have had different results. Finally, most studies included in this review were conducted in high-risk populations. Studies in a low- or moderate-risk group may reveal different results.

A final point we would like to address is an urgent need for increasing awareness among pediatricians for placental lesions and neonatal outcome. The obstetrician sends the placenta to the pathologist for histological examination. The results of the examination are reported back to the obstetrician. In most cases the pediatrician is unaware of the results of the placental examination. In the light of the accumulating evidence, however, that placental pathology is associated with perinatal mortality, neonatal morbidity, and neurological outcome, pediatricians should make an effort to obtain the results of placental examinations. Placental pathology, ascending intrauterine infection, and fetal thrombotic vasculopathy in particular, may help to identify the group of neonates at risk of adverse neonatal outcome. Monitoring these infants more closely could be helpful. Knowledge of the pathophysiological mechanisms leading to neonatal mortality and morbidity may lead the way to finding early intervention strategies to improve infants' morbidity and outcome.

## Supporting Information

Checklist S1 **PRISMA flowchart of identified articles published between January 1995 and October 2013.**
(DOC)
